# Snakebite Management: The Need of Reassessment, International Relations, and Effective Economic Measures to Reduce the Considerable SBE Burden

**DOI:** 10.1007/s44197-024-00247-z

**Published:** 2024-06-10

**Authors:** Ramesh Kumar, Anurag S. Rathore

**Affiliations:** Department of Chemical Engineering, Indian Institute of Technology Delhi, Hauz Khas, New Delhi, India

**Keywords:** Anti-snake venom, Anti-snake venom manufacturing, Export and import, Global policy, PROMISE approach, Snakebite envenoming management

## Abstract

The sole treatment for snakebite envenomation (SBE), the anti-snake venom (ASV), suffers from considerable drawbacks, including side effects and limited species specificity. Additionally, despite its existence for more than a century, uniform availability of good quality ASV does not yet exist. The present review describes the journey of a SBE victim and highlights the global crisis of SBE management. A detailed analysis of the current ASV market has also been presented along with the worldwide snake distribution. The current production of country specific licensed ASV throughout the globe along with their manufacturers has been examined at the snake species level. Furthermore, a detailed analysis of on-ground situation of SBE management in antivenom manufacturing countries has been done using the most recent literature. Additionally, the export and import of different ASVs have been discussed in terms of procurement policies of individual countries, their shortcomings, along with the possible solution at the species level. It is interesting to note that in most countries, the existence of ASV is really either neglected or overstated, implying that it is there but unsuitable for use, or that it is not present but can be obtained from other countries. This highlights the urgent need of significant reassessment and international collaborations not just for development and production, but also for procurement, distribution, availability, and awareness. A PROMISE (Practical ROutes for Managing Indigenous Snakebite Envenoming) approach has also been introduced, offering simple, economical, and easy to adopt steps to efficiently alleviate the worldwide SBE burden.

## Introduction

Anti-snake venom (ASV) is the only approved treatment for snakebite envenoming (SBE), the biggest public health crisis, that arises when a snake injects poisonous venom following a bite [1–3]. Despite the availability of ASVs for more than 100 years, more than 150,000 people are likely to die from snakebites globally, especially the poor in rural areas in developing countries [2, 4, 5]. The World Health Organization (WHO), in its 2019 and 2021 roadmaps, has aimed to halve the annual global SBE burden by 2030 [6, 7]. One of the four pillars of this roadmap is availability of safe, effective, and economical ASVs to people, and its improved control and regulation [8]. However, there have been reports about the flooding of market with low quality ASVs following the cessation of production by a major global ASV manufacturer [9]. The complexity of the venom components, geographical limitations, low funding, and improper ASV regulation are other issues in SBE management [4, 10, 11]. The latter is particularly important since even countries with capabilities of ASV production fail to properly manage SBE without proper ASV distribution policies. Thus, to reduce SBE burden and to ensure that no victim is left without treatment, it is necessary to understand the ASV market for better disbursement, regulation, and regional control. This will help in bilateral improvements in health policies with direct benefit to the poor victims and marked reduction in SBE induced fatalities and disability adjusted life years.

The present review summarizes the major causes of global crisis in SBE management and current ASV manufacturing practices of all the recognized ASV producers in a single map at the species level. To the best of our knowledge, this is the highest resolution snake biodiversity and antivenom manufacturing map ever published. For countries that do not produce, but import ASVs, respective import routes and procurement flaws have been highlighted, and alternative vendor countries have been suggested in terms of species specific ASVs availability. Finally, a PROMISE (Practical ROutes for Managing Indigenous Snakebite Envenoming) approach has also been proposed comprising of straightforward, cost-effective, and easily implementable measures for immediate reduction in the global SBE burden.

This article is expected to aid policymakers for improved fund allocation, regulation of ASV production, procurement, and distribution, with learning from case studies of several countries around the world. This information should also be useful for decision making for countries who wish to procure ASV from alternative countries that produce more specific ASVs. Additionally, it will aid in educating the physicians about the ASVs available in one’s own and neighboring countries and improved SBE patient management. Finally, the PROMISE approach should serve as easy to implement ideas for any country interested in reducing SBE burden without significant economical investments.

## Method

Initial search was done on Scopus with the term ‘snakebite’ or ‘antivenom’ along with individual ‘country name’ in ‘article title, abstract, and keyword’ section. Region specific studies from 2020 onwards that documented the on-ground situation of antivenom availability in the country were included. Studies focused on alternative antivenoms, patient case studies, venom characterizations, non-human victims, other animals, and antivenom manufacturing were excluded. In case no relevant article could be found on Scopus, a general internet search was done using the same keywords to find the relevant literature. Where appropriate, the reference list of included articles was also screened to identify the most appropriate article.

## The Global SBE Management Crisis

**Fig. 1:**
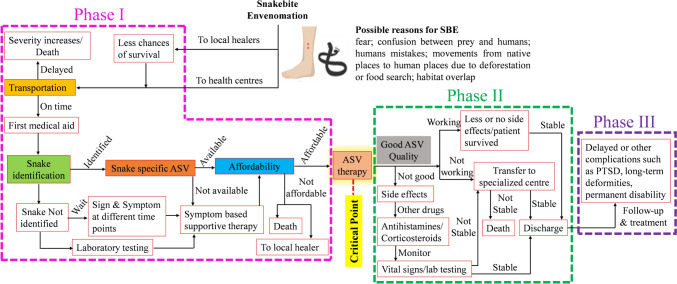
The journey of a SBE victim. The coloured boxes indicate the major hurdles in ASV therapy. The outcome of the antivenom therapy can be lifesaving or fatal depending upon the quality, specificity, efficacy, dosage, time of administration, and side effects of the antivenom administered. Note that affordability includes ASV cost as well as other hospital expenses

A victim’s journey from SBE to receive ASV therapy is filled with multiple hurdles (Fig. 1). The first is transportation to a health centre because SBE is largely the disease of the poor people from rural or remote areas in developing or low-income countries. These places often lack good health care centres and have poor transportation facilities which also force patients to resort to traditional healers over modern medicine [12–14]. Nevertheless, once a patient manages to reach a health care center, the next hurdle is SBE identification, because not all snakes present with a distinct clinically identifiable set of symptoms, the snake description reported by the patient or the attendant is often inadequate, and many health care practitioners (HCPs) have only limited knowledge about SBE [2, 15]. Other hurdles include availability of the species-specific and good quality ASV in appropriate quantity. Affordability is another major hurdle, not just that the ASVs are expensive, but the costs of additional therapies and hospital further limits affordability for the poor [16]. For a patient who crosses all these hurdles, the administration of the ASV serves as a critical life-saving point and marks the end of, what we call, the phase I of the patient’s journey to SBE therapy.

The administration of only a safe, affordable, specific, and good quality ASV can mitigate the effects of SBE [17]. This is the beginning of phase II of the patient’s post therapy struggles because the administration of the ASV does not guarantee permanent cure. Low quality ASV can lead to mild to severe side effects in patients [18]. Further, ASV may not always be effective in preventing SBE mediated damage. This is often the case when the patient arrives hours after the bite or the ASV administration is delayed because of phase I hurdles. Studies recommend early ASV administration within 2–6 h post bite [19–21]. This is because delayed ASV therapy can prevent further damages, but it cannot reverse the damage already done [22]. Additionally, delay in bite to needle time not only increases systemic envenomation, but also increases the number of ASV vials required for treatment, along with length of hospital stay, as well as the risk of morbidity and mortality [19].

Unfortunately, even after administration of a good quality and effective ASV, complete cure from SBE effects is not guaranteed and late adverse reactions of ASV and disability adjusted life years are common [2, 18]. This is phase III of the patient struggle which may continue for the entire lifespan. Nevertheless, the earliest possible administration of safe and specific ASV is the only available treatment paradigm to save a SBE victim’s life and this is still not accessible in many countries. A deeper analysis about the targeted species, ASV production, and procurement strategies is thus needed to fully understand the global status of ASVs.

## The Global Status of Anti-Snake Venoms

**Fig. 2:**
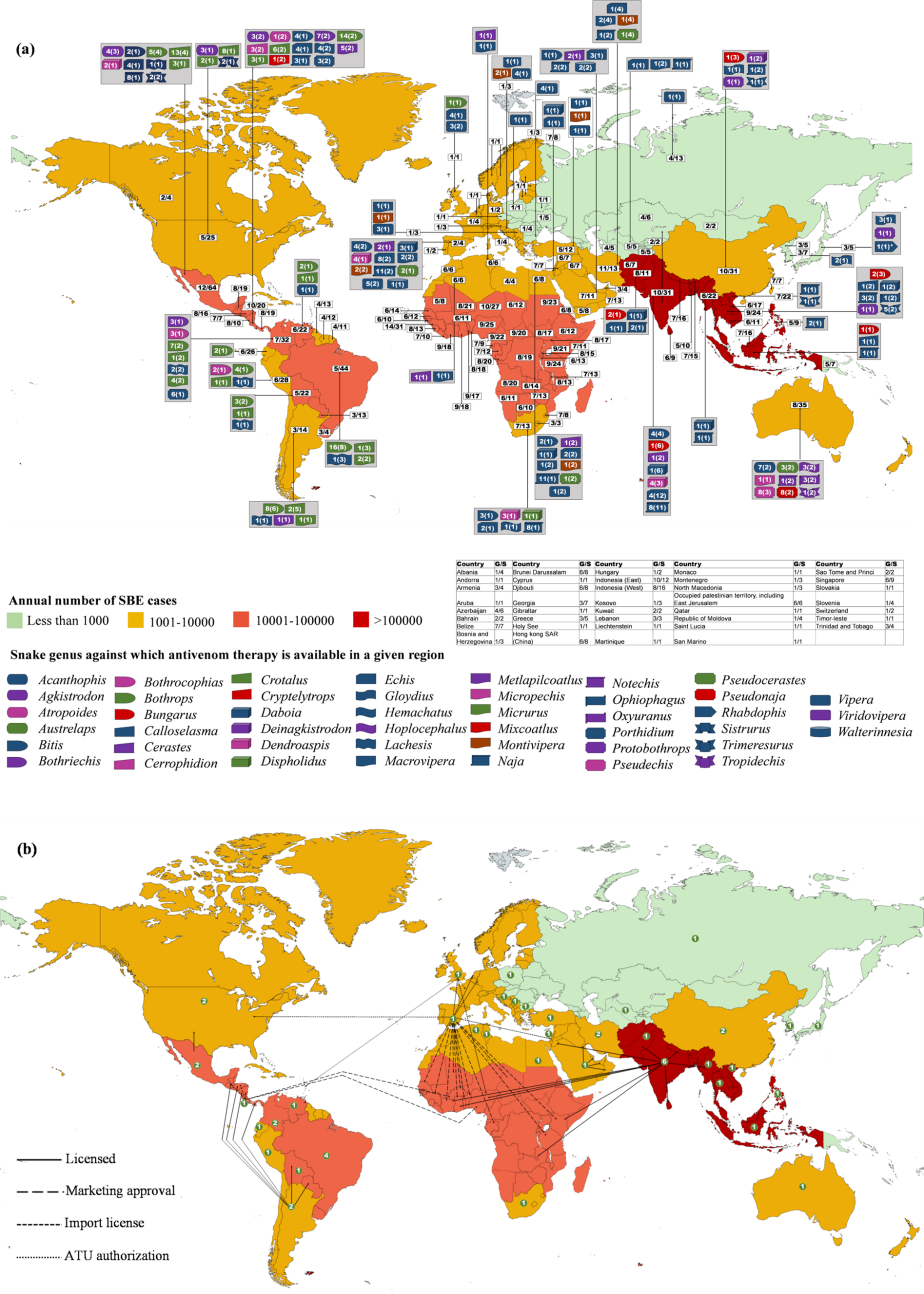
Geographical distribution of snakes, snakebites, anti-snake venom production, the targeted genera, and species. **a** Global snakebite cases, snake distribution, ASV production, its distribution, and the targeted genera. Numbers in white boxes represent the number of snake genera followed by number of species found in respective countries. Each symbol represents a genus targeted by the antivenom(s) produced in each country. The number of targeted species of a genus is written inside the corresponding symbol. Numbers in parentheses represent the total number of antivenoms produced by the manufacturing country against the given genus. * represents investigational product. G/S correspond, respectively, to the number of genera and species present in a country. Note: Ecuador shut down ASV manufacturing in 2012, and the last batch of Croatia ASV expired in 2019. Both countries are now dependent on imports [24] [25]. **b** Antivenom manufacturers and import/export. Numbers inside the circles represent the number of antivenom manufacturers in the corresponding country. Note: Kenya recalled Indian antivenoms in 2023 due to inefficacy and is aiming self-manufacturing [26] [27]. Based on published data and WHO [23, 28]

Although SBE continues to be a cause of worldwide concern, it has affected some parts of the world worse than others (Fig. 2). The global distribution of SBE burden, number of snake genera and species found in each country, ASV production in various countries, targeted genera, and the number of anti-snake venoms manufactured against each genus in different regions, as listed in the WHO database [23], are summarized in Fig. 2a. The corresponding export and import network of anti-snake venoms along with the number of ASV manufacturers in each country are presented in Fig. 2b.

The number of ASV manufacturers have increased from 46 to 51 since 2018 [23, 29]. However, the variability in the global ASV distribution and the limited availability of ASV against some genera is still quite evident. Furthermore, the ASV against many genera are produced by a single manufacturer in a given country. In fact, 29 out of 37 countries have a single ASV manufacturer. A detailed description of the ASV status around the world is discussed below.

### Asia

This region has the highest burden of SBE in the world and contributes to 70% SBE mortality globally [30]. There are about 300 snake species including 70 venomous snakes. However, most countries manufacture ASV against only a few species. As a result, the use of non-specific ASVs for treating bites from non-targeted species is frequent across almost all countries. For example, in mainland China, envenomations with green pit viper are recommended to be treated with ASVs against snakes from the same subfamily Crotalinae with unsatisfactory therapeutic efficacy [31]. Even in India, which is a leading producer and exporter of ASVs in the world, ASVs against the big four species is used to treat all SBE cases. However, this polyvalent ASV is ineffective in treating bites from other medically important snake species present in the region, such as *H.hypnale*, *Echis sochureki*, *Naja kaouthia*, and *C. malabaricus*, voicing the need for availability of appropriate ASVs [32–35]. Also, the Indian polyvalent ASV exhibits limited efficacy against identical species in different parts of the country due to differences in the venom composition [32, 36–38].

Of the many manufacturing countries, India and Iran are the only ASV exporters in the region. While the Iranian ASV is permitted in a few Middle East countries, India exports its ASV to Africa, Middle East, and South Asia. It can be said that the South Asian region is majorly dependent on the Indian ASVs. Nevertheless, since South Asia bears 70% of the global SBE burden, special attention must be given to the Indian ASV manufacturers [30]. No information about the ASVs available in Bangladesh and Afghanistan could be found in the WHO database despite the significantly high SBE burden in these regions.

### Africa

In Africa, a region with a high annual SBE incidence of up to 100 thousand, most afflicted countries do not produce ASV. The continent has only four ASV manufacturers listed in the WHO database, one each in Algeria, Tunisia, Egypt, and South Africa. Antivenoms from neither of these 4 manufacturers is approved in other African countries. The sub-Saharan Africa region, which bears the second largest burden of SBE after Asia, thus has only one ASV producer in this region i.e. South Africa [9]. Here also, there is discrepancy in ASV availability between rural and urban areas [39]. The African countries depend on imports from other continents, which risks the supply chain and raises the cost of already expensive ASVs which is estimated to be US$100–153 in the region [9]. Further, the market is unstable and the production of many ASVs has been stopped in the past. Chad, Ghana, Central Africa Republic, and Nigeria have already faced increased mortality due to the poor quality of some ASVs when a leading foreign ASV manufacturer stopped supply [9]. Furthermore, efficacy data of the imported ASVs is majorly lacking [40]. In addition, the venoms used for ASV production are sourced from limited suppliers only. Given the vastness of this region and the biogeographical venom variation of the multitude of species found here [41], extensive cross-reactivity studies of existing ASVs can be an interim solution to the SBE problem.

### North America

There are more than 30 species of pit vipers in the region and almost 9,000 SBE cases are reported annually [42]. Within the family, rattlesnakes account for the majority of the casualties because of their potent venom and widespread distribution. The first ASV was approved as early as 1954. Costa Rica is the major ASV producer in the area followed by Mexico and USA, in terms of number of targeted snake species. Costa Rica is also a major exporter of ASVs and has product marketing approvals in neighbouring as well as African countries.

### South America

Manufacturing of ASVs is an old tradition since 1901 when the first ASV was developed in Brazil [43]. However, only limited amounts of ASV are produced due to the restricted capacities of the only four ASV producing laboratories [44]. Antivenom is also produced in Argentina, Peru, Colombia, Venezuela, and Bolivia. However, despite the presence of multiple ASV manufacturers, many countries are dependent on import and ASV supply is usually insufficient [45]. Also, not all the ASVs are undergo quality control and preclinical efficacy assessments [43]. The importance of the former can be understood from Eucador, where ASV manufacturing was decommissioned owing to flaws in production process, and the manufacturing plant was closed in 2012 [24]. Ecuador is now dependent on ASV import from Costa Rica.

### Europe

It has a burden of approximately 8000 SBE annually and the medically relevant venomous snakes belong to the Viperinae family [46, 47]. There is a long history of ASV production in Europe and almost 13 laboratories produced it in early 1980s. However, in Croatia, the last ASV batch expired in 2019 and Croatia, despite possessing ASV manufacturing capabilities, is currently dependent on imports [25]. Nevertheless, trust in immunotherapy, has recently increased with availability of purified and well tolerated ASVs. There are several ASVs for vipers in Europe [47, 48]. Recently, a new polyvalent ASV has been shown to be effective against European vipers [49]. Only two manufacturers from United Kingdom and Spain export ASVs. The Spanish ASV is a cornerstone product for many African countries. This is a Pan-African ASV which is claimed to target 22 snake species from 4 genera. However, this product has not been recommended by the WHO. The WHO has terminated its risk–benefit assessment without making any recommendations [23]. Also, none of the European ASVs is licensed by the European Medical Agency [48].

### Australia

Snakebite envenomings are rare in Australia, but some cases may be life-threatening [50]. Brown snake, tiger snakes, black snakes, death adder, taipan, and sea snakes are the major clinically important snakes in the region [51]. ASV is produced in the region since 1930 and ASVs were developed against all major snake groups as early as 1962 [52]. However, there is only a single manufacturer, and it does not export its ASVs. Commercial snake venom detection tests are available, however, due to the polyvalent nature of Australian ASVs and problems with the kit's results, the emphasis has turned to identifying envenoming in patients rather than snake identification [53]. The region also has diverse species of sea snakes but a single monovalent ASV, raised against a Malaysian snake, is used to treat all sea snake envenomations [21].

Despite the aforementioned issues, different countries of the world have made attempts to lower the SBE burden in a variety of ways, but a number of barriers still exist. Table 1 summarizes the most recent information on these.

**Table 1:** Effective steps and barriers to SBE management

Region	Country^a^	Manufacturer(s)	Effective steps	Barriers in SBE management	References
Asia	China	National Institute of Preventive Medicine Shanghai Serum Bio-technology Co Ltd		Haphazard ASV supply Non-specific ASVs ASV shortage	[31]
	India	Biological E Limited Premium Serums and Vaccines Pvt. Ltd VINS Bioproducts Ltd King Institute of Preventative Medicine and Research Haffkine Biopharmaceutical Corporation Ltd Bharat Serums and Vaccines Limited	Emergency ambulance service with lifesaving equipment and drugs, including ASV	Under reporting of SBE and mortality Lack of safe and effective antivenoms Poor healthcare facilities Inclination towards traditional healers ASV manufactured only against big four Untrained medical staff	[91–94]
	Indonesia	PT Bio Farma (Persero)	Antivenom cross-neutralization data required for marketing approval SBE treatment costs covered on co-payment basis	A single ASV is available Costly and limited ASV Antivenom ineffective against many Indonesian species Absence of antivenom in nearby health care facilities and lack of transportation Absence of cold-chain storage Cultural barriers	[75, 95]
	Iran	Razi Vaccine & Serum Research Institute Padra Serum Alborz	Toxicology trained physicians National unified protocol for SBE management	Under-reporting of SBE cases Preference of traditional treatment No formal clinical trial of one antivenom Antivenom starting dose not established by formal clinical trial Wound incision and fasciotomy still practiced	[74]
	Israel	Kamada limited	Short distance to hospital in some areas Uncertain snake identification in many cases	Lack of uniform treatment protocol for SBE No antivenom available against one of the venomous endemic species-*Atractaspis engaddensis*	[96, 97]
	Japan	KM Biologics Co. Ltd	Snake institute to help physicians	Unapproved antivenom against *R. tigrinus*	[98]
	Myanmar	Myanmar pharmaceutical factory	Myanmar Snakebite Project Antivenom usage reported to ministry	lack of pharmaceutical logistic system affects antivenom distribution	[75]
	Pakistan	National Institute of Health		Inclination towards traditional healers Long transportation times Manufactures liquid antivenom but has poor refrigeration facilities Low domestic supply No guidance protocol for antivenom production Inadequately trained health care workers	[99]
	Philippines	Biologicals Manufacturing Division (Research Institute for Tropical Medicine)	Subsidised antivenom production SBE treatment cost included in health insurance	Seek traditional healers ASV shortage Ineffective ASV supply chain	[75]
	Republic of Korea	KoreaVaccine Co Ltd	National reference standard for antivenom	Costly antivenoms Validated guideline for antivenoadministration unavailable No ASV against *R. tigrinus*	[100–102]
	Saudi Arabia	National Antivenom & Vaccine Production Center (NAVPC)	Good medical facility Antivenom available even in remote areas Established national records		[103]
	Thailand	Queen Saovabha Memorial Institute	Well-established supply chain real-time antivenom inventory	No national training on SBE management since 2016	[75]
	Vietnam	Institute of Vaccines and Biological Substances (IVAC)		Inclination towards traditional practices Poor documentation of SBE records Lack of SBE statistics Limited studies on clinical presentation of SBE No national protocol for SBE management Antivenom shortage and high cost Long distance to hospitals Lack of trained HCPs Lack of SBE education and public awareness Monovalent antivenoms prevalent which require accurate snake identification Use of antivenoms of unknown efficacy and potency Only one study on adverse reaction to antivenom so far	[68]
Africa	South Africa	South African VaccinProducers (SAVP)	Free toxicology advice to HCPs and public at all hours Trained physicians Free snake identification charts	Discrepancy in antivenom availability in urban and rural areas SBE reporting not mandatory Need of cold chain transport Short expiry of ASVs	[39, 104]
	Tunisia	Institut Pasteur de Tunis		No validated scale of SBE severity Limited studies on SBE Lack of health facilities in rural areas Delay between bite and hospital arrival Propensity for at home first aid such as tourniquets	[105]
North America	Costa Rica	Instituto Clodomiro Picado	Free antivenom SBE notification mandatory Traditional medicine rarely used Quality control of antivenoms Good cold chain National protocol for SBE diagnosis and treatment Regular training sessions on SBE management	A part of population is devoid of SBE related governmental aids, such as agricultural workers Access to health facility delayed in some regions	[106]
	Mexico	Birmex (Instituto Nacional de Higiene) Laboratorios Silanes, S. A. de C. V		Inclination towards traditional healers Limited data on SBE epidemiology	[107]
	United States	BTG International Inc Wyeth (owned by Pfizer)		Cost transparency Concerns about insurance cover Antivenom not available at all facilities Costly antivenom Controversial maintenance therapy	[108, 109]
South America	Bolivia	Ministerio de Salud y Deportes, Instituto Nacional de Laboratorios De Salud		Snake misidentification Untrained HCPs	[110]
	Brazil	Fundacao Ezequiel Dias (FUNED) Centro de Producao e Pesquisas de Immunobiol Instituto Butantan Instituto Vital Brazil S.A	Compulsory case notification Free antivenom	Inclination towards traditional practices No antivenom in rural areas Lack of trust in local health care Low confidence among clinicians in SBE management Overdosing and under-dosing of antivenoms Antivenom expiration owing to lack of inventory Antivenom storage issues due to lack of stable electricity Limited ASV production capacity	[64, 111]
	Colombia	Instituto Nacional de Salud (CO) Laboratorios Biologicos PROBIOL Ltda	Mandatory reporting	Regular antivenom shortage Lack of cold chain transport No policy for antivenom distribution	[63]
	Ecuador	Instituto Nacional de Higiene y Medicina Tropical "Leopoldo Izquieta Pérez"	Fixed maximum price for antivenom	Antivenom production stopped in 2012 Dependent on import of ASV from Costa Rica	[24]
Europe	Croatia	Imunološki Zavod (Institute of Immunology)	Low reported mortality	Last antivenom batch expired in 2019 Dependent on imports now	[25]
	Serbia	Institute of Virology, Vaccine and Sera TORLAK	Antivenom available Snakes protected by law	HCPs unawareness of species in their areas Snake misidentification common	[85]
	Spain	INOSAN BIOPHARMA S. A. (Spain)	Very few cases and fatalities		[112]
	The United Kingdom	Micropharm Ltd	24 h rapid clinical advice available through poison centres	Exotic snakebites a challenge	[113]
Australia	Australia	Seqirus Pty Ltd		Costly ASV Need of cold chain transport Limited shelf-life Single antivenom to treat envenoming by all sea snakes – due to rarity of sea snake envenoming-more time for administering antivenom than terrestrial SBE cases Limited clinical evidence to support use of this antivenom for sea snake envenoming	[21]

The table lists the anti-snake venom (ASV) manufacturing countries and companies. Additionally, it presents a non-exhaustive list of effective steps taken by ASV manufacturing countries and the existing gaps in snakebite management. The information has been sourced from the WHO snake ASV database [23] and the most recent literature (2020-present) to provide insights into the present situations in these countries. The list is by no means exhaustive and additional steps or gaps might be present in the respective countries

^a^For Algeria, Argentina, Bulgaria, Egypt, Peru, Poland, Russia, Turkey, Uzbekistan, and Venezuela, no relevant literature, published 2020 onwards, could be found

## The Problem with Imported Anti-Snake Venoms

It is important to remember that, even when ASVs from one nation are used to treat SBE in another, the ASV may not be successful because of regional differences, leading to less effective ASVs on the market, as seen in the case of Sri Lanka [54–56]. Also, there are cases where the imported ASVs are not raised against any endemic species of a country. For example, in Jordan, one of the countries in middle east with maximum number of snakebites, the single imported ASV is raised against African snakes that do not occur in Jordan and is clinically ineffective [57]. Analysis of the efficacy of imported ASVs against envenomation by endemic species is important as their efficacy is not always guaranteed. Recently, Kenya recalled the Indian ASV upon evidence of its inefficacy in treating Kenyan snake envenomation cases [26]. It must be noted that the recalled ASV is listed in the WHO snake ASV database and is claimed to target many species found in Kenya [23]. Kenya may now partner with Costa Rica for technology transfer and aim local ASV production in next three years, as per the reports [27]. However, bringing Kenyan ASV to market will not be an easy task. Sri Lanka had earlier collaborated with Costa Rica and US for a similar goal [22]. However, it has been almost a decade since the collaboration, but the clinical trial data has not been published. In their another collaboration with a leading antivenom manufacturer in India, a product has shown better pre-clinical efficacy than currently used Indian ASV, however, its additional pre-clinical and clinical studies are still required [58]. The country is still dependent on import from India, whose ASV has been reported to bear limited efficacy against Sri Lankan snake species [54, 58].

For almost all countries, the imported ASVs do not target all regional species. Some of these missed species are even of the highest medical importance with their respective ASVs available for sale in other countries. The list of possible vendor countries where ASV is available for each of the missed species of each importing country (as per WHO database) is provided in Table 2. Organizations like WHO that deal in the purchase and distribution of ASVs in underdeveloped nations like sub-Saharan Africa should also find Table 2 useful [8]. For example, the Spanish ASVs may be useful in targeting species of Jordan and the Brazilian ASV for targeting snakes in Paraguay. Greater international cooperation between countries is thus needed to import other/additional ASVs. Still, for many other species no ASV is available in any part of the world. Production of newer ASVs, inclusion of venoms of these species in immunogen mixtures used for ASV production, or cross-reactivity studies of existing ASVs should be undertaken for dealing envenomation cases with these species.

**Table 2:** The global export–import network of anti-snake venom (ASV)

Region	Importing country	Exporting country	Antivenom name	Claimed species targeted in importing country	Missed species [possible vendor countries]	Missed species—no antivenom available
Asia	Israel	Spain	Inoserp™ MENA	*Cerastes cerastes, Cerastes gasperettii, Daboia palaestinae, Echis coloratus, Pseudocerastes fieldi, Walterinnesia aegyptia*	None	*Atractaspis engaddensis, Montivipera bornmuelleri*
	Israel	Israel	Vipera palaestinae Antiserum (Equine source)	*Daboia palaestinae*		
	Israel	Israel	Echis coloratus Antiserum (Equine source)	*Echis coloratus*		
Asia	Jordan	India	Snake Venom Antitoxin (Menaven)	*None*	*Cerastes gasperettii* [Spain, Saudi Arabia], *Daboia palaestinae* [Egypt, Israel, Spain], Echis coloratus [Egypt, Israel, Saudi Arabia, Spain, The United Kingdom], *Macrovipera lebetina* [Algeria, Croatia, Egypt, Iran (Islamic Reublic of), Spain, Serbia, Tunisia, Turkey, Uzbekistan], *Pseudocerastes fieldi* [Egypt, Spain], *Walterinnesia aegyptia* [Saudi Arabia, Spain]	*Atractaspis engaddensis*
			Snake Venom Antiserum (Echis)			
Asia	Kuwait	Saudi Arabia	Polyvalent Snake Antivenom—Equine	*Cerastes gasperettii, Walterinnesia morgani*	None	None
Asia	Myanmar	India	Snake Venom Antiserum I.P. (Asia)	None	*Bungarus candidus* [Thailand], *Bungarus fasciatus* [Indonasia, Thailand], *Naja siamensis* [Thailand], *Naja sumatrana* [Thailand], *Ophiophagus hannah* [Thailand], *Protobothrops mucrosquamatus* [China], *Trimeresurus albolabris* [Thailand, Vietnam], *Trimeresurus erythrurus* [Thailand], *Trimeresurus purpureomaculatus* [Thailand]	*Bungarus bungaroides, Bungarus niger, Bungarus flaviceps, Bungarus magnimaculatus, Bungarus wanghaotingi, Naja mandalayensis, Protobothrops jerdonii, Protobothrops kaulbacki, Trimeresurus gumprechti, Trimeresurus guoi, Trimeresurus yunnanensis*
	Myanmar	Myanmar	Anti-Viper (Russell's viper)	*Daboia siamensis*		
	Myanmar	Myanmar	Siamese cobra antivenin	*Naja kaouthia*		
Asia	Nepal	India	Snake Venom Antiserum I.P. (Asia)	*Bungarus caeruleus,Daboia russelii, Naja naja*	*Bungarus fasciatus* [Indonasia, Thailand], *Naja kaouthia* [Myanmar, Thailand, Vienam], *Ophiophagus hannah* [Thailand]	*Bungarus bungaroides, Bungarus lividus, Bungarus niger, Bungarus walli, Gloydius himalayanus, Protobothrops jerdonii, Protobothrops himalayanus, Trimeresurus salazar, Trimeresurus septentrionalis, Trimeresurus tibetanus*
Asia	Oman	Saudi Arabia	Polyvalent Snake Antivenom—Equine	*Bitis arietans, Cerastes gasperettii, Echis coloratus, Echis omanensis, Naja arabica*	*Echis carinatus* [India, Iran (Islamic Republic of), Pakistan, Spain, Uzbekistan], *Echis khosatzkii* [Spain], *Pseudocerastes persicus* [Iran (Islamic Republic of), Spain], *Vipera ammodytes* [Bulgaria, Croatia, Egypt, Serbia, Spain, Turkey], *Vipera berus* [Bulgaria, Croatia, Poland, Russian Federation, Serbia, Spain, The United Kingdom], *Vipera ursinii* [Bulgaria, Croatia]	*Atractaspis andersonii, Vipera nikolskii*
Asia	Pakistan	India	Snake Venom Antiserum I.P. (Asia)	*Bungarus caeruleus, Daboia russelii, Echis carinatus, Naja naja*	*Macrovipera lebetina* [Algeria, Croatia, Egypt, Iran (Islamic Reublic of), Spain, Serbia, Tunisia, Turkey, Uzbekistan], *Pseudocerastes persicus* [Iran (Islamic Republic of), Spain]	*Bungarus persicus, Eristicophis macmahonii, Gloydius himalayanus*
	Pakistan	Pakistan	Polyvalent Antisnake Venom Serum	*Bungarus caeruleus, Bungarus sindanus, Daboia russelii, Echis carinatus, Naja naja, Naja oxiana*		
Asia	Qatar	Saudi Arabia	Polyvalent Snake Antivenom—Equine	*Cerastes gasperettii*	None	None
Asia	Sri Lanka	India	Snake Venom Antiserum I.P. (Asia)	*Bungarus caeruleus, Daboia russelii, Echis carinatus, Naja naja*	None	*Bungarus ceylonicus, Hypnale hypnale, Hypnale nepa, Hypnale zara, Trimeresurus trigonocephalus*
Asia	UAE	Saudi Arabia	Polyvalent Snake Antivenom—Equine	*Cerastes gasperettii, Echis omanensis*	*Echis carinatus* [India, Iran (Islamic Republic of), Pakistan, Spain, Uzbekistan], *Pseudocerastes persicus* [Iran (Islamic Republic of), Spain]	None
Africa	Mali	Spain	Inoserp™ Pan-Africa	*Bitis arietans, Dendroaspis polylepis, Echis leucogaster, Echis ocellatus, Echis pyramidum, Naja haje, Naja katiensis, Naja nigricollis, Naja pallida, Naja savannula, Naja senegalensis, Naja subfulva*	*Cerastes cerastes* [Algeria, Egypt, India, Saudi Arabia, Spain, Tunisia], *Dispholidus typus* [South Africa]	*Atractaspis fallax, Atractaspis micropholis, Atractaspis watsoni, Echis hughesi, Echis jogeri,Naja ashei, Thelotornis mossambicanus*
Africa	Morocco	Spain	Inoserp™ MENA	*Bitis arietans, Cerastes cerastes, Daboia mauritanica, Echis leucogaster, Naja haje*	None	*Vipera monticola*
Africa	Nigeria	Costa Rica	EchiTAb-plus-ICP Liquid	*Bitis arietans, Bitis gabonica, Bitis nasicornis, Echis leucogaster, Echis ocellatus, Echis romani*	*Dispholidus typus* [South Africa], *Naja katiensis* [Spain], *Naja senegalensis* [Spain]	*Atheris broadleyi, Atheris chlorechis, Atheris squamigera, Atractaspis irregularis, Atractaspis micropholis, Atractaspis watsoni, Pseudohaje goldii, Pseudohaje nigra, Thelotornis kirtlandii*
	Nigeria	India	Snake Venom Antiserum (Africa)	*Bitis arietans, Bitis gabonica, Dendroaspis jamesoni, Dendroaspis viridis, Echis leucogaster, Echis ocellatus, Naja haje, Naja melanoleuca, Naja nigricollis, Naja savannula, Naja subfulva*		
	Nigeria	The United Kingdom	EchiTAbG	*Echis ocellatus, Echis romani*		
Africa	Republic of Congo	Mexico	Antivipmyn® Africa^a^	*Bitis arietans, Bitis gabonica, Dendroaspis polylepis, Naja haje, Naja melanoleuca, Naja nigricollis, Naja subfulva*	*Bitis nasicornis* [Costa Rica, India, Spain], *Dendroaspis jamesoni* [India, Spain, South Africa], *Dispholidus typus* [South Africa]	*Atheris broadleyi, Atheris squamigera, Atractaspis bibronii, Atractaspis irregularis, Naja anchietae, Naja annulata, Naja christyi, Pseudohaje goldii, Thelotornis kirtlandii, Thelotornis capensis*
Africa	Sierra Leone	Spain	Inoserp™ Pan-Africa	*Bitis arietans, Bitis nasicornis, Bitis rhinoceros, Dendroaspis polylepis, Dendroaspis viridis, Naja guineensis, Naja nigricollis, Naja savannula*	*Dispholidus typus* [South Africa]	*Atheris chlorechis, Echis jogeri, Pseudohaje nigra, Thelotornis kirtlandii*
Africa	Senegal	Spain	Inoserp™ Pan-Africa	*Bitis arietans, Dendroaspis polylepis, Dendroaspis viridis, Echis leucogaster, Echis ocellatus, Naja katiensis, Naja nigricollis, Naja savannula, Naja senegalensis*	*Dispholidus typus* [South Africa]	*Atractaspis microlepidota, Atractaspis micropholis, Atractaspis watsoni, Echis jogeri*
Africa	Tanzania	Spain	Inoserp™ Pan-Africa	*Bitis arietans, Bitis gabonica, Bitis nasicornis, Dendroaspis angusticeps, Dendroaspis jamesoni, Dendroaspis polylepis, Naja haje, Naja nigricollis, Naja pallida, Naja subfulva*	*Dispholidus typus* [South Africa], *Naja mossambica* [Costa Rica, Egypt, South Africa]	*Atheris squamigera, Atractaspis bibronii, Atractaspis fallax, Atractaspis irregularis, Naja annulata, Naja ashei, Pseudohaje goldii, Proatheris superciliaris, Thelotornis capensis, Thelotornis kirtlandii, Thelotornis mossambicanus, Thelotornis usambaricus*
Africa	Togo	Spain	Inoserp™ Pan-Africa	*Bitis arietans, Bitis nasicornis, Bitis rhinoceros, Dendroaspis jamesoni, Dendroaspis viridis, Echis ocellatus, Naja guineensis, Naja katiensis, Naja nigricollis, Naja savannula, Naja senegalensis*	*Dispholidus typus* [South Africa]	*Atheris chlorechis, Atractaspis irregularis, Atractaspis watsoni, Pseudohaje nigra, Thelotornis kirtlandii*
Africa	Gabon	Spain	Inoserp™ Pan-Africa	*Bitis arietans, Bitis gabonica, Bitis nasicornis, Dendroaspis jamesoni, Naja melanoleuca, Naja nigricollis*	None	*Atheris broadleyi, Atheris squamigera, Atractaspis irregularis, Naja annulata, Pseudohaje goldii, Thelotornis kirtlandii*
Africa	Ghana	India	Snake Venom Antiserum (Africa)	*Bitis arietans, Dendroaspis jamesoni, Dendroaspis viridis, Echis ocellatus, Naja guineensis, Naja nigricollis, Naja savannula*	*Dispholidus typus* [South Africa]	*Atheris chlorechis, Atractaspis irregularis, Atractaspis watsoni, Pseudohaje goldii, Pseudohaje nigra, Thelotornis kirtlandii*
	Ghana	Mexico	Antivipmyn® Africa^a^	*Bitis arietans, Dendroaspis viridis, Echis ocellatus, Naja guineensis, Naja nigricollis, Naja savannula*		
	Ghana	Spain	Inoserp™ Pan-Africa	*Bitis arietans, Bitis nasicornis, Bitis rhinoceros, Dendroaspis jamesoni, Dendroaspis viridis, Echis ocellatus, Naja guineensis, Naja katiensis, Naja nigricollis, Naja savannula, Naja senegalensis*		
Africa	Guinea	Spain	Inoserp™ Pan-Africa	*Bitis arietans, Bitis gabonica, Bitis nasicornis, Bitis rhinoceros, Dendroaspis jamesoni, Dendroaspis polylepis, Dendroaspis viridis, Echis ocellatus, Naja guineensis, Naja katiensis, Naja melanoleuca, Naja nigricollis, Naja savannula, Naja senegalensis*	*Acanthophis laevis* [Australia], *Acanthophis rugosus* [Australia], *Dispholidus typus* [South Africa], *Micropechis ikaheka* [Australia], *Oxyuranus scutellatus* [Australia], *Pseudechis papuanus* [Australia], *Pseudechis rossignolii* [Australia], *Pseudechis rossignolii* [Australia]	*Atheris chlorechis, Atheris squamigera, Atractaspis irregularis, Atractaspis micropholis, Echis jogeri, Naja annulata, Pseudohaje goldii, Pseudohaje nigra, Thelotornis kirtlandii*
Africa	Ivory Coast	Spain	Inoserp™ Pan-Africa	*Bitis arietans, Bitis nasicornis, Bitis rhinoceros, Dendroaspis polylepis, Dendroaspis viridis, Echis ocellatus, Naja guineensis, Naja katiensis, Naja nigricollis, Naja savannula, Naja senegalensis*	*Dispholidus typus* [South Africa]	*Atheris chlorechis, Atractaspis irregularis, Atractaspis micropholis, Pseudohaje goldii, Pseudohaje nigra, Thelotornis kirtlandii*
Africa	Zambia	India	Snake Venom Antiserum (Africa)	*Bitis arietans, Bitis gabonica, Dendroaspis polylepis, Naja nigricollis, Naja subfulva*	*Dispholidus typus* [South Africa], *Naja annulifera* [South Africa], *Naja mossambica* [Costa Rica, Egypt, South Africa]	*Atractaspis bibronii, Naja annulata, Naja anchietae, Thelotornis capensis, Thelotornis kirtlandii, Thelotornis mossambicanus*
Africa	Benin	Mexico	Antivipmyn® Africa^a^	*Bitis arietans, Bitis gabonica, Dendroaspis polylepis, Dendroaspis viridis, Echis leucogaster, Echis ocellatus, Naja melanoleuca, Naja nigricollis, Naja savannula*	*Dispholidus typus* [South Africa]	*Atractaspis micropholis, Atractaspis watsoni, Pseudohaje nigra, Thelotornis kirtlandii*
		Spain	Inoserp™ Pan-Africa	*Bitis arietans, Bitis gabonica, Bitis nasicornis, Dendroaspis jamesoni, Dendroaspis polylepis, Dendroaspis viridis, Echis leucogaster, Echis ocellatus, Naja katiensis, Naja melanoleuca, Naja nigricollis, Naja savannula, Naja senegalensis*		
Africa	Burkina Faso	Costa Rica	EchiTAb-plus-ICP Liquid	*Bitis arietans, Echis leucogaster, Echis ocellatus, Naja nigricollis*	*Dispholidus typus* [South Africa]	*Atractaspis micropholis, Atractaspis watsoni*
	Burkina Faso	Spain	Inoserp™ Pan-Africa	*Bitis arietans, Dendroaspis polylepis, Echis leucogaster, Echis ocellatus, Naja katiensis, Naja nigricollis, Naja savannula, Naja senegalensis*		
Africa	Cote d'Ivoire	India	Snake Venom Antiserum (Africa)	*Bitis arietans, Dendroaspis polylepis, Dendroaspis viridis, Echis ocellatus, Naja guineensis, Naja nigricollis, Naja savannula*	*Bitis nasicornis* [Costa Rica, India, Spain], *Bitis rhinoceros* [Costa Rica, India, Spain], *Dispholidus typus* [South Africa], *Naja katiensis* [Spain], *Naja senegalensis* [Spain]	*Atheris chlorechis, Atractaspis irregularis, Atractaspis micropholis, Pseudohaje goldii, Pseudohaje nigra, Thelotornis kirtlandii*
Africa	Kenya	India	Snake Venom Antiserum (Africa)	*Bitis arietans, Bitis gabonica, Dendroaspis jamesoni, Dendroaspis polylepis, Naja haje, Naja nigricollis, Naja subfulva*	*Dispholidus typus* [South Africa]	*Thelotornis mossambicanus, Atheris desaixi, Atheris squamigera, Atractaspis bibronii, Atractaspis fallax, Atractaspis irregularis, Naja ashei, Pseudohaje goldii, Thelotornis usambaricus*
	Kenya	Spain	Inoserp™ Pan-Africa	*Bitis arietans, Bitis gabonica, Bitis nasicornis, Dendroaspis angusticeps, Dendroaspis jamesoni, Dendroaspis polylepis, Echis pyramidum, Naja haje, Naja nigricollis, Naja pallida, Naja subfulva*		
Africa	Chad	Spain	Inoserp™ MENA	*Bitis arietans, Cerastes cerastes, Echis leucogaster, Naja haje, Naja nigricollis, Naja nubiae*	*Dispholidus typus* [South Africa], *Echis romani* [Costa Rica, India, Mexico, The United Kingdom], *Naja savannula* [Egypt, India, Mexico, Spain, South Africa], *Naja subfulva* [Egypt, India, Mexico, Spain, South Africa]	*Atractaspis micropholis, Atractaspis watsoni,*
North America	Guatemala	Costa Rica	PoliVal-ICP Lyophilized	*Agkistrodon bilineatus, Bothriechis bicolor, Bothriechis schlegelii, Bothriechis thalassinus, Bothrops asper, Cerrophidion godmani, Crotalus simus, Metlapilcoatlus mexicanus, Metlapilcoatlus occiduus, Metlapilcoatlus olmec, Porthidium nasutum, Porthidium ophryomegas*	*Bothriechis aurifer* [Colombia], *Crotalus tzabcan* [Mexico], *Micrurus nigrocinctus* [Costa Rica, Mexico]	*Agkistrodon russeolus, Micrurus browni, Micrurus diastema, Micrurus elegans,*
	Guatemala	Argentina	Suero Antiofidico Centroamericano BIOL	*Bothrops asper, Crotalus simus*		
North America	Honduras	Argentina	Suero Antiofidico Centroamericano BIOL CLB	*Bothrops asper, Crotalus simus*	None	*Agkistrodon howardgloydi, Bothriechis guifarroi, Cerrophidion wilsoni, Micrurus alleni, Micrurus diastema*
	Honduras	Costa Rica	PoliVal-ICP Liquid	*Bothriechis marchi, Bothriechis schlegelii, Bothriechis thalassinus, Bothrops asper, Crotalus simus, Metlapilcoatlus indomitus, Metlapilcoatlus mexicanus, Metlapilcoatlus occiduus, Porthidium nasutum, Porthidium ophryomegas*		
	Honduras	Costa Rica	CoRal-ICP Liquid	*Micrurus nigrocinctus*		
	Honduras	Costa Rica	PoliVal-ICP Lyophilized	*Bothriechis marchi, Bothriechis schlegelii, Bothriechis thalassinus, Bothrops asper, Crotalus simus, Metlapilcoatlus indomitus, Metlapilcoatlus mexicanus, Metlapilcoatlus occiduus, Micrurus nigrocinctus, Porthidium nasutum, Porthidium ophryomegas*		
North America	Martinique	The United Kingdom	BothroFAV	*Bothrops lanceolatus*	None	None
North America	Nicaragua	Argentina	Suero Antiofidico Centroamericano BIOL CLB	*Bothrops asper, Crotalus simus*	None	*Agkistrodon howardgloydi, Cerrophidion wilsoni, Micrurus alleni*
	Nicaragua	Costa Rica	PoliVal-ICP Liquid	*Bothriechis schlegelii, Bothrops asper, Crotalus simus, Metlapilcoatlus mexicanus, Porthidium nasutum, Porthidium ophryomegas*		
	Nicaragua	Costa Rica	PoliVal-ICP Lyophilized	*Bothriechis schlegelii, Bothrops asper, Crotalus simus, Metlapilcoatlus mexicanus, Porthidium nasutum, Porthidium ophryomegas*		
	Nicaragua	Costa Rica	CoRal-ICP Liquid	*Micrurus nigrocinctus*		
North America	Panama	Argentina	Suero Antiofidico Centroamericano BIOL CLB	*Bothrops asper*	*Bothriechis nigroviridis* [Colombia], *Micrurus mipartitus* [Colombia]	*Micrurus alleni, Bothrops punctatus, Cerrophidion sasai, Micrurus clarki, Micrurus dissoleucus, Micrurus dumerilii*
	Panama	Costa Rica	PoliVal-ICP Liquid	*Atropoides picadoi, Bothriechis lateralis, Bothriechis schlegelii, Bothriechis supraciliaris, Bothrops asper, Lachesis acrochorda, Lachesis stenophrys, Metlapilcoatlus mexicanus, Porthidium lansbergii, Porthidium nasutum*		
	Panama	Costa Rica	PoliVal-ICP Lyophilized	*Atropoides picadoi, Bothriechis lateralis, Bothriechis schlegelii, Bothriechis supraciliaris, Bothrops asper, Lachesis acrochorda, Lachesis stenophrys, Metlapilcoatlus mexicanus, Porthidium lansbergii, Porthidium nasutum*		
	Panama	Costa Rica	CoRal-ICP Liquid	*Micrurus nigrocinctus*		
North America	USA	Mexico	Anavip®	*Agkistrodon contortrix, Agkistrodon piscivorus, Crotalus adamanteus, Crotalus atrox, Crotalus horridus, Crotalus molossus, Crotalus oreganus, Crotalus ruber, Crotalus scutulatus, Crotalus viridis, Sistrurus miliarius*	None	*Crotalus cerberus, Crotalus lepidus, Crotalus ornatus, Crotalus pricei, Crotalus pyrrhus, Crotalus stephensi, Crotalus tigris, Crotalus willardi, Micruroides euryxanthus, Sistrurus tergeminus*
		Spain	Inoserp™ MENA	*Cerastes cerastes*		
			Inoserp™ Pan-Africa	*None*		
	USA	USA	CroFab (Crotalidae Polyvalent Immune Fab (Ovine))	*Agkistrodon contortrix, Agkistrodon piscivorus, Agkistrodon taylori, Crotalus adamanteus, Crotalus atrox, Crotalus horridus, Crotalus molossus, Crotalus oreganus, Crotalus ruber, Crotalus scutulatus, Crotalus viridis, Sistrurus catenatus, Sistrurus miliarius*		
	USA	USA	North American Coral Snake Antivenin (Equine)	*Micrurus fulvius, Micrurus tener*		
South America	Bolivia	Argentina	Suero Antiofidico Polivalente BIOL	*Bothrops diporus, Crotalus durissus*	*Bothrocophias hyoprora* [Peru], *Bothrocophias microphthalmus* [Peru], *Bothrops bilineatus* [Brazil, Colombia], *Bothrops brazili* [Brazil, Costa Rica, Peru], *Bothrops mattogrossensis* [Brazil, Colombia, Mexico]*, Bothrops moojeni* [Argentina*,* Brazil, Costa Rica], *Bothrops taeniatus* [Brazil], *Micrurus lemniscatus* [Brazil, Colombia], *Micrurus spixii* [Colombia], *Micrurus surinamensis* [Colombia]	*Bothrocophias andianus, Bothrops sanctaecrucis, Micrurus annellatus, Micrurus hemprichii, Micrurus narduccii, Micrurus obscurus, Micrurus pyrrhocryptus*
	Bolivia	Bolivia	Suero Antiofidico Polivalente Botropico/Crotalico	*Bothrops atrox, Bothrops jonathani,Bothrops neuwiedi, Crotalus durissus*		
	Bolivia	Bolivia	Suero Antiofidico Polivalente Botropico/Laquesico	*Bothrops atrox, Bothrops neuwiedi, Lachesis muta*		
South America	Paraguay	Argentina	Suero Antiofidico Polivalente BIOL	*Bothrops alternatus, Bothrops diporus, Crotalus durissus*	*Bothrops jararaca* [Argentina, Brazil, Colombia, Costa Rica], *Bothrops jararacussu* [Argentina, Brazil, Costa Rica], *Bothrops mattogrossensis* [Brazil, Colombia, Mexico], *Bothrops moojeni* [Argentina, Brazil, Costa Rica], *Bothrops pubescens* [Brazil], *Micrurus corallinus* [Argentina, Brazil], *Micrurus lemniscatus* [Brazil, Colombia]	*Micrurus altirostris, Micrurus frontalis, Micrurus pyrrhocryptus*
Europe	Belgium	Spain	Inoserp™ MENA	None	*Vipera berus* [Bulgaria, Croatia, Poland, Russian Federation, Serbia, Spain, The United Kingdom]	None
			Inoserp™ Pan-Africa	None		
Europe	Cyprus	Spain	Inoserp™ Pan-Africa	None	*Macrovipera lebetina* [Algeria, Croatia, Egypt, Iran (Islamic Republic of), Spain, Serbia, Tunisia, Turkey, Uzbekistan]	None
Europe	France	Spain	Inoserp™ Pan-Africa	None	*Vipera ursinii* [Bulgaria, Croatia]	*Vipera seoanei*
		The United Kingdom	ViperaFAV	*Vipera aspis, Vipera berus*		
Europe	Germany	Spain	Inoserp™ Pan-Africa	None	*Vipera aspis* [Bulgaria, Croatia, Serbia, Spain, The United Kingdom], *Vipera berus* [Bulgaria, Croatia, Poland, Russian Federation, Serbia, Spain, The United Kingdom]	None
Europe	Netherlands	Spain	Inoserp™ MENA	None	*Vipera berus* [Bulgaria, Croatia, Poland, Russian Federation, Serbia, Spain, The United Kingdom]	None
			Inoserp™ Pan-Africa	None		
Europe	United Kingdom	Spain	Inoserp™ MENA	None	None	None
			Inoserp™ Pan-Africa	None		
		The United Kingdom	EchiTAbG	None		
			ViperaFAV	*Vipera berus*		
			ViperaTAb®	*Vipera berus*		

The claimed species lists the species present in the respective country and claimed to be targeted by a given ASV as per the WHO database [23]. The missed species list the snake species present in a country which are not targeted by any antivenom available in the respective country. The countries which manufacture antivenom(s) against these missed species are mentioned in parentheses. The missed species for which no antivenom is manufactured in any part of the world are listed under ‘missed species-no antivenom available’

^a^Registration expired

## Lessons from the World-The PROMISE Approach

**Fig. 3:**
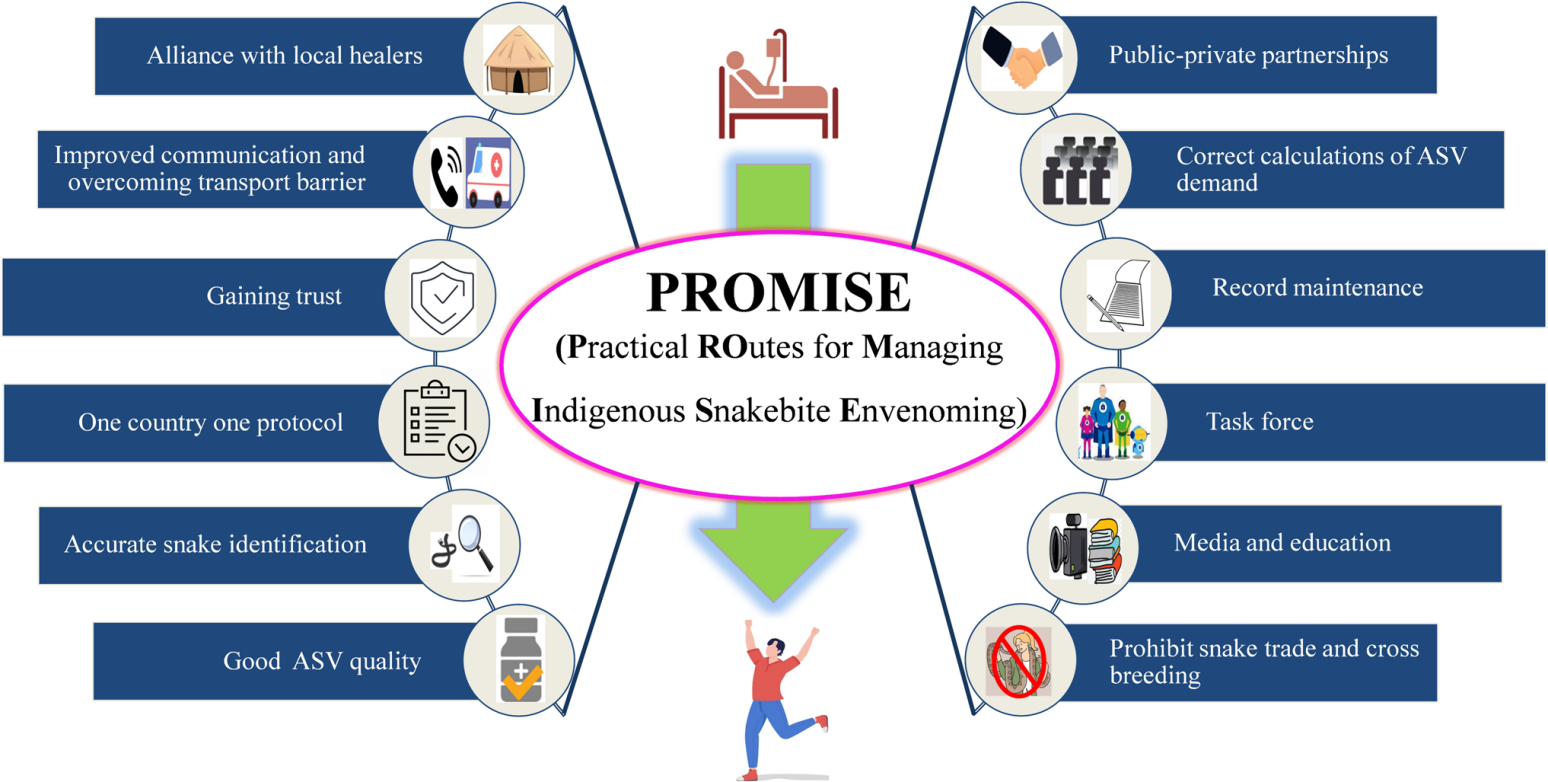
The PROMISE approach for SBE management. The PROMISE (Practical ROutes for Managing Indigenous Snakebite Envenoming) approach is an amalgamation of economical and easy remedies whose implementation can significantly reduce the SBE burden

Now that we have established that ASV administration is the crucial part of the SBE treatment process, it is obligatory to investigate the current ASV market. Nevertheless, other obstacles also play a key role while treating SBE (Fig. 1 and Table 1). Although infrastructure improvements and research into developing next generation ASVs [59] are definitely needed to ensure availability of ASVs in distant areas and overcoming limitations of current ASVs, as also discussed in other roadmaps [60], these are not expected to be encouraged given the neglected nature of SBE. Thus, to improve the other aspects of SBE management, we recommend the PROMISE (Practical ROutes for Managing Indigenous Snakebite Envenoming) approach which comprises of economical and simple yet effective steps to be undertaken by all countries interested in reducing SBE in their regions. The approach is discussed below in detail and summarized in Fig. 3.

### Alliance with Local Healers

The integration of traditional healers into the SBE management pathway should be considered. In this regard, a registry of legitimate healers must be made and advertised among the masses. While the registry would ensure the exclusion of non-qualified self-claimed healers who can worsen patient’s condition, the release of a list of authorized healers from the regulatory agencies would help in gaining the trust of those local people who prefer traditional therapists over hospital care. The HCPs can teach these registered local healers about the initial SBE management and local healers may help with diagnosis, first aid, and timely referral to health care centres. Besides saving lives, their referrals would also help in estimating SBE statistics more accurately and reduce under-reporting. Success with such an integrated approach has been reported in African countries for Human African Trypanosomiasis (sleeping sickness), HIV, and psychosis in terms of improved diagnosis or clinical outcomes for patients [14, 61].

### Improved Communication and Overcoming Transport Barrier

Improvements in phone and internet services, and transportation can enhance the health seeking nature among the masses, as in Ethiopia [62]. The transportation barrier can also be partially lifted by facilitating ASV distribution and establishment of poison information centers. In absence of a centralized ASV distribution program based on local SBE epidemiology, concentration of ASVs in populated areas over others and increased product cost may occur, as in Colombia [63]. Also, victims in rural and remote areas may remain deprived of ASV despite in-house production and free distribution, such as in Brazil [64]. Further, strict vigilance is needed for adequate distribution of ASVs across all levels of healthcare systems. In Kenya, ASV is unavailable in dispensaries despite governmental guidelines [65]. Additionally, the teleconsultation services of poison information centers can also prevent patient transport, help in knowledge propagation among clinicians, and potentially lower expenses and expedite diagnosis and treatment, such as in Taiwan [66]. The guidelines for establishing such centres have recently been updated by the WHO [67]. Also, their establishment should also be followed by its publicity among the HCPs. In South Africa, many physicians do not call such centres simply because they do not know that such centres exist [39].

### Gaining Trust

#### Patients in Healthcare System

In Brazil, even if some rural areas have access to ASVs, people prefer to travel long distances in a search for more trustworthy treatment [64]. This is one important issue which is usually ignored by most policy makers. A potential solution could involve the circulation of annual reports from healthcare centers regarding SBE cases treated and their corresponding outcomes, both within the healthcare community and to the public.

#### Doctors in ASV Therapy

The trust of doctors on ASV therapy is also important. In a survey conducted in Vietnam, doctors advocated the superiority of traditional healers over ASV for SBE management [68]. Also, in Kenya, health care workers have reported non-administration of available ASV to victims because of fear of adverse reactions [69]. Sharing case reports of successful ASV therapy on a regular basis can be beneficial in this regard.

#### Doctors in Themselves

Treatment of SBE is not included in the medical course syllabus even in countries with high SBE burden such as India [15, 70]. Further, due to the least number of victims or the available treatments, usually the preparedness of the health workers towards SBE is minimum. In multiple surveys as well, HCPs from different countries have admitted their lack of knowledge in SBE management [15]. Management of SBE and ASV associated adverse reactions must thus be included in the curriculum of undergraduate medical students. Also, the clinicians should be regularly updated about SBE case classification and management through training programs. Simultaneously, educating the HCPs about dry bites, that are snakebites without venom injection, is also paramount [71]. A survey in a Saudi Arabia University revealed that while medical students received training for SBE management, around 80% were unaware of dry bites [72].

### One country One Protocol

A national unified protocol for SBE management should be prepared to reduce ASV usage, treatment cost, and also the duration of hospital stay, as in case of Iran [73, 74]. The protocol reduced the ASV usage by 4 vials, treatment cost by $196, and hospital stay by 1 day per patient. The existing protocols of neighboring countries and the treatment guidelines for SBE by the WHO might serve as a reference for preparing such national protocols. For countries like India, which have a wide variety of snake species and different qualities and cross-reactivities of antivenoms, it is advisable to implement more rigorous quality control measures and modify the procedure to accommodate the specific snake diversity of each region. Alternatively, it may be beneficial to develop protocols that are specific to individual states or regions.

### Accurate Snake Identification

An inventory of regional snakes, available ASVs and their targeted species, and an SBE management chart should be available in every treating facility, especially in the rural areas. This information must be available in local languages to ensure understanding of common masses. This will aid in correct snake identification and boost the confidence of both patients and HCPs in SBE management. For example, the use of a snake atlas, as in Ethiopia, can be a simple yet powerful tool in this regard [62]. The non-venomous species must also be documented in the snake atlas to minimize the confusion and the associated unnecessary treatment or side effects. If possible, a real-time inventory should be made such as in Thailand, to map required ASV availability in nearby areas [75]. Additionally, HCPs must be made aware of the online databases, such as the WHO snakebite platform, WCH clinical toxicology, and iNaturalist, which can be used not only for self-education but also for accurate snake identification with the patient attendants to facilitate their recall with the snake pictures available in the database [23, 76, 77]. Mobile application based databases are also available. One such application is’SnakeHub’ which is a free mobile app that provides description of 114 snake species found in Kerala, India in English as well as in local language [78]. Efforts must also be made for developing mobile applications for image based correct snake identification such as those using artificial intelligence and SnakeSnap app [79, 80]. When creating such apps, one can contact local rescuers for assistance. Adequate awareness about such apps must be spread among both the HCPs as well as common masses for improving snake identification.

Another way of aiding clinicians in ASV selection, improving therapeutic efficacies of SBE treatment and reducing mortality is the development of venom detection kits [81]. However, efforts must be made to ensure that the kits are economical and cause a minimum increment in the cost of ASV therapy. Further, the sensitivity and specificity of such kits must be thoroughly tested. Currently, despite availability of venom detection kits, the Australian clinicians are focusing more on diagnosing envenoming rather than the kit results [53].

### Good ASV Quality

Only Good Manufacturing Practices (GMPs) and stringent quality control can ensure the production of a safe and effective ASV [17]. However, sudden imposition of stringent regulations or reforms, after years of neglect, should be avoided. This important lesson can be learnt from Ecuador, a country with ASV manufacturing history since 1981, which shut down ASV manufacturing following sudden imposition of GMPs and identification of other production flaws [24]. Winning the manufacturer’s confidence, for example, by providing time and incentives for upgrading production practices, should be a priority to ensure a sustainable approach for effective implementation of governmental policies.

### Public–Private Partnerships

The onus to prove ASV specificity lies on the manufacturer, but it can be accelerated with public–private partnerships. The governments or the regulatory authorities should also partner with the manufacturers in a search for expanding the ASV cross-reactivity to other endemic species found in the country. Collaborations with academia may also be promoted in this regard, since full expertise might not be available under a single roof. Thus, while efficacy data would ensure that the imported ASV would be clinically suitable, the cross-reactivity studies would expand the customer’s base. Such studies offer a promising approach to decrease the burden of SBE while newer ASVs are being developed. In many studies, using a mouse model, a single ASV was shown to exhibit cross-reactivity against several heterologous medically significant snakes from sub-Saharan Africa [82, 83]. However, certain conditions must be met before using these cross-neutralization test-passed ASVs against heterologous venoms. For example, the disparities in metabolic activity between mice and humans call for additional clinical testing [84]. Additionally, such studies should include long-term assessment for toxicities of different venom components. For example, neutralizing the paralytic effects of *N. sumatrana* venom in mice for 24 h does not guarantee that the hematoxic or other effects will be neutralized, and the animals will survive [84].

Further, the manufacturers must be encouraged to stay updated about the recent advancements in ASV production and incorporate appropriate changes in their production processes to generate broadly specific, affordable, safe, and effective (BASE) ASVs [17].

### Correct Calculations of ASV Demand

The strategy for calculating ASV demand needs also be finalized in such a way that access to ASV is guaranteed. As reported in a Colombian study, the different methods used to calculate ASV usage can yield widely varying results [63]. While one method, based on the theoretical average number of ASV vials used for treatment, suggested a yearly need of 25,380 vials, another method based on accident severity suggested a need of 50,021 vials in the country. Yet another method, that used multiple variables, such as underreporting and number of accidents in the previous decade, suggested the need of 54,440 vials. In addition to doing reasonable calculations, it is imperative to have contingency plans to handle unexpected surges in ASV demand, particularly during emergency situations like floods that may result in an increase in snakebite cases.

### Record Maintenance

Mandatory reporting of snakebites will not only help in better understanding of the regional SBE epidemiology, but also aid in estimating the actual ASV demand, better snake identification, and decision making for ASV administration. This can be achieved with manuals that include details about SBE case presentation, diagnosis, treatment, and clinical course description along with the name and amount of ASV administered. The regional records can also be combined into a centralized data bank accessible to public, physicians, researchers, and policymakers for effective SBE management. Extra care must also be taken when entering the species name. Incorrect snake name entries in medical publications can be a cause of concern [85]. It is also advisable to integrate pictures of the biting species wherever feasible.

### Task Force

A task force comprising of frontline healthcare professionals should be established to conduct surveillance and collect data on occurrences of SBE, mortality and socio-economic burden. Such studies, even if done to cover only a fraction of population, as underway in India, can be valuable in managing the distribution of ASV and for better governmental policies such as fund allocation for SBE management [86]. It must be noted that the choice of protocol can affect the estimates of SBE burden. For example, utilizing disability weights determined from the community, rather than those derived from the global burden of disease (GBD), can lead to more accurate estimations of SBE burden [87]. Furthermore, protocols must be created in compliance with international guidelines such as the STROBE guidelines for cross-sectional studies [88].

### Media and Education

The media should exclusively distribute accurate information in a responsible manner. For example, in Serbia, reports regarding the occurrences of even the non-venomous snakes and rare bites, have been reported to be dramatized by media [85]. A role reversal of the media, as also mentioned by the author, can help in disseminating accurate knowledge among the masses about SBE and its treatment. Another simple strategy to educate the masses can be the introduction of SBE management in the school curriculum. Alternatively, annual workshops can be conducted by schoolteachers for the same. Another approach can be ‘A Snake A Month’ (ASAM) session, where school children are taught about one regional snake every month.

### Prohibit Snake Trade and Cross Breeding

The trend of rearing venomous snakes by individuals results in increased number of SBE [25, 89]. Even in countries, such as Brazil, where snake trading is prohibited, illegal ownership of venomous snakes has been reported [90]. In Switzerland, where private housing of snakes is legal, physicians are often untrained in dealing patients envenomed by exotic pets [89]. Similarly, the cross-breeding of snakes to create hybrids of related snake species should be strictly banned, since the hybrids have unknown venom composition and ASV efficacy in bitten victims cannot be guaranteed [89].

## Conclusions

The present study provides a comprehensive review about the gaps in SBE therapy, current status of ASV manufacturing, global distribution, and the economical remedies to provide a holistic approach for SBE management. For an SBE victim, the acquisition of ASV demands a painful journey with multiple hurdles with unsure outcomes. The critical point in this journey is the administration of a safe and effective ASV, which is often not available due to the uneven distribution of SBE burden and ASV manufacturing across the world with the latter being limited to a few countries. Even in countries with ASV production abilities, ASVs are produced against a few species only and only a few manufacturers export their ASVs to other countries. Furthermore, the quality of the imported ASVs is not guaranteed and some countries even import ASVs against species that are not even found in their country, despite the availability of specific anti-snake venoms in other countries. A self-reassessment for the currently imported ASVs and enhanced support for ASV supply is thus needed at the international level. Also, although significant advancements have been made in the strategies for ASV production, it is still underproduced and is in short supply in different countries. Many countries, especially in Africa, do not have approval for imported ASVs despite the high SBE burden. Consequently, there is a huge imbalance between ASV demand and supply and the need for ASV is far more than estimated. We thus call for a reassessment of the global ASV demand, procurement and distribution policies, and enhanced responsibility of both manufacturers and importers for ASV quality. Also, urgent attention is needed towards ASV manufacturing and/or cross-reactivity studies for species for which no ASV is available. Additionally, a PROMISE approach, comprising several simple and economical steps have been proposed in this article that can be taken to overcome the hurdles to SBE management without major costs. The findings of this study could also serve as a ready reference for the clinicians and policy makers who want to educate themselves about the ASVs available in their country, and neighboring areas.

## Data Availability

This published article contains all the generated and analyzed data, thus no additional data source is required.
